# Bayesian Networks for Clinical Decision Support in Lung Cancer Care

**DOI:** 10.1371/journal.pone.0082349

**Published:** 2013-12-06

**Authors:** M. Berkan Sesen, Ann E. Nicholson, Rene Banares-Alcantara, Timor Kadir, Michael Brady

**Affiliations:** 1 Department of Engineering Science, University of Oxford, Oxford, United Kingdom; 2 Faculty of Information Technology, Monash University, Clayton, Victoria, Australia; 3 Mirada Medical, Oxford, United Kingdom; 4 Department of Oncology, University of Oxford, Oxford, United Kingdom; University of Torino, Italy

## Abstract

Survival prediction and treatment selection in lung cancer care are characterised by high levels of uncertainty. Bayesian Networks (BNs), which naturally reason with uncertain domain knowledge, can be applied to aid lung cancer experts by providing personalised survival estimates and treatment selection recommendations. Based on the English Lung Cancer Database (LUCADA), we evaluate the feasibility of BNs for these two tasks, while comparing the performances of various causal discovery approaches to uncover the most feasible network structure from expert knowledge and data. We show first that the BN structure elicited from clinicians achieves a disappointing area under the ROC curve of 0.75 (± 0.03), whereas a structure learned by the CAMML hybrid causal discovery algorithm, which adheres with the temporal restrictions, achieves 0.81 (± 0.03). Second, our causal intervention results reveal that BN treatment recommendations, based on prescribing the treatment plan that maximises survival, can only predict the recorded treatment plan 29% of the time. However, this percentage rises to 76% when partial matches are included.

## Introduction

The accelerating trend towards personalised medicine, in parallel with the rapid development of various machine learning (ML) tools, has triggered the utilisation of medical datasets to propose diagnostic and prognostic options, to the point even of recommending individualised treatment plans [1,2]. In the context of clinical decision support (CDS), ML tools are used to assist the clinicians arrive at more informed treatment decisions based on past patient records. Such systems typically operate by matching a patient record to the information ‘learned’ from past patient records for which prescribed treatment plans and patient outcomes are known.

Medical datasets are usually characterised by their incompleteness and noisiness, which cause a substantial level of uncertainty while processing them [2]. Overall, uncertainty permeates causality in medicine, although it is not always made explicit. For example, in a dataset that contains ‘Age’ and ‘Survival’, the causal relationship between the two is evident even though it may not be straightforward to pinpoint through which variables it may be established. More importantly, uncertainty also arises naturally in patient care processes that underlie the data, not least in questions such as: “What is the probability of survival for this patient?” and “How do different treatment decisions affect this probability?” 

A prime example of a clinical setting, in which uncertainty is ubiquitous, is treatment selection in cancer care, where the diverse nature of the patient and disease characteristics and the rapidly expanding range of treatment options often present dilemmas regarding optimum treatment decisions [3]. As a consequence of the complex and inter-disciplinary nature of the decision making process, treatment plans for cancer patients are managed in multidisciplinary team (MDT) meetings that mobilise the joint expertise of clinicians from different specialisations. 

Personalised survival prediction and treatment selection are prominent in the MDT environment. Predicting the answer to the first of the above questions relates to prognostic reasoning [4]. An accurate prediction of survival can be used to stratify cancer patients into different risk groups and potentially aid in devising personalised treatment plans [5,6]. Furthermore, predicted survival information can also be pivotal in managing patient and family expectations on treatment outcomes [7]. As a probabilistic expression, this prognostic question may be denoted “P(Survival=Alive |Evidence)= ?”. Using a BN, this question can be answered via observational inference, where the focus is on discovering the posterior distribution of the query variable: Survival, conditioned on the observed Evidence for other nodes.

On the other hand, the second question, which queries the effects of treatment selections on the prognostic outcome, addresses the pragmatic goal of curative cancer care. Naturally, if the prognosis for the patient is poor, the end goal may be palliation and management of symptoms, rather than increasing the likelihood of survival. In terms of probability theory, this query is denoted as “P(Survival=Alive |Evidence, T)= ?”, where T represents the treatment plan variable. Compared to the former, this query aims to find the posterior distribution of Survival conditioned on T, which is – unlike Evidence- unobserved at the time of asking the question. In other words, the question is hypothetical and cannot be answered simply by the values observed to that point. In order to predict what the survival probability is going to be, given different treatment options, we would need to make a causal intervention, which allows us to ask “What if?” questions. This type of causal reasoning is highly important in CDS applications and is not compatible with discriminative ML methodologies such as regression models [8,9].

## Bayesian Networks

BNs enable causally reasoning with domain concepts in a visually appealing and more intuitive fashion compared to many other ML techniques [9], and they can be used to address the above clinical questions. They encode uncertain domain knowledge in a natural manner. A BN consists of a directed acyclic graph (DAG), and an underlying joint probability distribution, which together provide a mathematically sound and compact way to encode uncertainty in a given domain. From the outset, medical informatics has been the main driver in the development of BNs [10,11]. This is partly due to their ability to intuitively encapsulate the causal links between the diagnostic or prognostic factors that are stored in medical datasets [4,12,13]. 

BNs are suitable tools for probabilistic inference that can aid clinical decision making, since 1) their graphical nature enables the information they contain to be easily understood by a clinician [14]; 2) they can formally incorporate prior knowledge while learning the structure and parameters of the network [15]; 3) they facilitate parameter estimation due to their compact representation of the joint probability space; 4) they not only allow observational inference but also causal interventions [9]; 5) they can be used to query any given node in the network and are therefore substantially more versatile compared to classifiers built based on specific outcome variables; and 6) they perform well in making predictions with incomplete data, since the predictor variables are used to estimate not only the query variable but also one another [16][5,17]. For a detailed coverage of BNs, the reader is referred to [9,13].

The primary motivation of this work is to investigate the feasibility of developing BNs in providing decision support for survival prediction and treatment selection in lung cancer care. Lung cancer is the leading cause of cancer-related mortality throughout the world [18][6]. Our analyses are based on an anonymised subset of the English Lung Cancer database (LUCADA), which includes more than 126,000 patients who were diagnosed between 2006 and 2010. We utilise this large and unique dataset to develop and evaluate a series of BNs whose structures are learned in turn by manual, automated and hybrid approaches. Structure learning of BNs remains something of a black art and therefore a secondary goal of the paper is to assess the suitability of different methodologies to uncover the causal structure of the domain using a real-life medical dataset of the size and complexity of LUCADA.

## Literature Review

Cruz and Wishart [19] report that the adoption of ML techniques for prognosis prediction and treatment selection is a relatively recent development. The existing literature on BNs and cancer mainly concerns applications to aid diagnosis, risk evaluation and survival prediction. Furthermore, among different cancer domains, there has been a concentration on applications in breast cancer [20–24] as compared to BN applications in other types of cancer [5,7,25–28]. 

In terms of relevant BN applications on survival prediction, in a study published in 2011, which aims to predict the 1-year life expectancy of 189 patients with skeletal metastases, Forsberg et al. achieved good predictive performance with an area under the ROC curve (AUC) of 0.83 [7]. In a more recent study based on a substantially larger dataset containing 146,248 patient records, Stojadinovic et al. built a BN to carry out personalised survival prediction for colon cancer, reporting an AUC value of 0.85 [16]. Neither of these studies compared the suitability of different approaches in the causal discovery of the domain structure. In addition, both causal interventions and the feasibility of treatment recommendations by the BNs were out of the scope of both studies. 

Focusing on lung cancer specific applications of BNs, in 2010 Jayasurya et al. designed a BN in order to predict survival in non-small cell lung cancer (NSCLC) patients treated with radiotherapy. They concluded that BN models achieve a higher predictive performance with missing data, compared to support vector machines and are therefore more suitable for the medical domain [5]. In a more technically oriented publication, Oh et al. proposed a BN structure learning algorithm that combined both physical and biological factors for predicting local failure in lung cancer [27]. However, both of these studies were based on datasets that contained limited numbers of patient records -for one study in [27] only 18 patients- necessitating replication on larger datasets. 

In summary, the number of studies reporting the application of BNs to cancer is limited. Furthermore, apart from a handful of exceptions, most published results are from preliminary studies based on limited patient data. To our knowledge, no prior work, which takes into account histological, clinical and demographic information based on a national dataset of the size of LUCADA, exists in survival prediction or treatment recommendation in lung cancer.

## Materials and Methods

The National Lung Cancer Audit (NLCA) has been collecting electronic patient data within the English Lung Cancer Database (LUCADA) since 2004. Through a data sharing agreement between the NLCA and the University of Oxford, we have had access to an anonymised subset of the LUCADA dataset in order to carry out research in the biomedical engineering fields of clinical decision support and machine learning. This dataset includes 126,986 English patient records entered into the system from the beginning of 2006 until the end of 2010. All potentially patient identifiable data were removed by the NLCA prior to making the data available.

Since LUCADA is collected primarily for audit purposes, it includes many administrative variables which are of tangential interest to this study. Based on the input of our clinical collaborators and the literature review, we focused our analyses on the 13 most commonly encountered LUCADA variables in the major national and international lung cancer care guideline documents [6,29–31]. In addition to their clinical relevance, these were selected on the basis of being available at the time a new patient is presented for a treatment decision to the MDT. These variables are listed in Table 1. 

**Table 1:** The 13 patient and disease specific variables from LUCADA, along with the values they can take and their temporal orders.

**Code**	**Name**	**Values**	**Temporal Tier**
1	Age	<50; 50-60; 60-70; 70-80; >80	Pre-treatment
2	Staging Identifier	6; 7	Pre-treatment
3	FEV1 Absolute Amount	<1.0; 1-1.5; 1.5 - 2.0; >2.0	Pre-treatment
4	FEV1 Percentage	<30; 30-40; 40-80; >80	Pre-treatment
5	Performance Status	0; 1; 2; 3; 4	Pre-treatment
6	Number of Comorbidities	0; 1; 2; 3; 4; 5	Pre-treatment
7	Primary Diagnosis	C33; C34; C34.0; C34.1; C34.2; C34.3; C34.8; C34.9; C38.4; C38.3; C38.8	Pre-treatment
8	Tumour Laterality	Left; Right; Midline; Bilateral; Not Applicable	Pre-treatment
9	TNM Category	IA; IB; IIA; IIB; IIIA; IIIB; IV; Uncertain	Pre-treatment
10	Histology	M8010/2; M8041/3; M8046/3; M8070/3; M8140/3; M8250/3; M8012/3; M8020/3; M8013/3; M8240; M8980/3; M8940/3; M9999/9	Pre-treatment
11	Site-specific Staging Classification	Limited; Extensive; Unknown	Pre-treatment
12	Suggested cancer treatment plan	Listed in Table 2	Treatment
13	1-yr Survival	Alive; Dead	Post-treatment

In Table 1, the first 11 variables are categorised as “pre-treatment variables”. They contain information about the patient or disease specific aspects of a patient record that are required before a treatment decision is made. Among the patient-related specifics listed: ‘Performance Status’ indicates general physical well-being, whereas ‘FEV1 Absolute Amount’ and ‘FEV1 Percentage’ store the lung capacity (more precisely, forced expiratory volume in 1 second) of a patient. In addition, ‘Number of co-morbidities’ provides information on the number of significant co-morbidities, such as cardiovascular disease and renal dysfunction, that a patient has at the time of diagnosis.

Among the disease specific variables, ‘Primary Diagnosis’ identifies the ICD-10 code [32] that best describes the location and the general type of the disease. ‘Histology’ indicates the SNOMED code [33] of the histo-pathological type of the primary tumour, and the American Joint Committee on Cancer (AJCC) defined ‘TNM category’ summarises the overall severity of the disease in terms of tumour size and spread of cancerous cells. Similarly, ‘Site-specific Staging Classification’ stores whether the disease is limited or extensive for small cell lung cancer patients. 

The ‘Suggested cancer treatment plan’ variable stores the treatment given to the patient. The definitive treatment for non-metastatic lung cancer is surgical resection. However, since most patients are only diagnosed when the disease is at an advanced stage, only 10-15 % of patients can be treated with surgery [34,35]. Table 2 lists all available treatment plan types within LUCADA, along with their frequencies. In this table, all treatment types, apart from Palliative Care (5) and Active Monitoring (6), are categorised as curative treatments. The treatments coded 1, 9, 10 and 11 are those that involve surgical resection. The rest of the treatments, coded 2, 3, 7 and 8, comprise individual chemotherapy and radiotherapy or a combination of the two.

**Table 2:** The available treatment plan options in LUCADA and their frequencies.

**Code**	**Name**	**Percentage (%)**
1	Surgery	10
2	Radiotherapy	14.79
3	Chemotherapy	19
5	Palliative care	23
6	Active Monitoring	9
7	Sequential chemotherapy and radiotherapy	7
8	Concurrent chemotherapy and radiotherapy	1
9	Induction chemotherapy to downstage before surgery	0.08
10	Neo-adjuvant chemotherapy and surgery	0.13
11	Surgery followed by adjuvant chemotherapy	2
-	Null	14

Finally, in Table 1, the ‘1-year survival’ variable contains the survival outcome information for all patient records. In cancer care, the 5-year survival rate is the most commonly used cut-off point to measure disease-free survival. Since LUCADA does not yet contain much patient data on 5-year survival, we use 1-year survival as a surrogate outcome measure. This choice was supported both by our clinical collaborators and by the literature, which reports almost all improvement in lung cancer survival as being attributable to an increase in 1-year survival [36,37]. The overall ‘1-year survival’ rate within LUCADA is 33%.

### Pre-processing the LUCADA dataset

Before designing a set of domain-specific BNs, we first analysed and pre-processed the LUCADA dataset. Data pre-processing is a crucial step in any machine learning exercise, since the reliability of a predictive model depends crucially on the quality of data used [38]. For this purpose, we carried out the following pre-processing steps.

First, we manually removed those records where the patient was diagnosed with Mesothelioma, since our focus was on NSCLC and small cell lung cancer (SCLC) patients. In addition, we removed those patient records for which the recorded treatment plan was Brachytherapy (less than 100 patients, making it unlikely) or there was no 1-year survival information. These deletions reduced the number of observations available in the dataset from 126,987 to 117,426.

Second, we discretised the “*Age*”, “FEV1 Percentage” and “FEV1 Absolute amount” data fields, which are the only non-categorical fields in the LUCADA dataset. While it is possible to build BNs with continuous variables, the majority of clinical applications to date utilise categorical variables [4]. These three variables were discretized based on clinician advice and on the cut off values given in the guideline rules. Although there are various techniques for automatic discretisation of continuous variables [39–41], the availability of cut-off values within the guideline documents and clinical collaborator advice enabled us to perform manual discretisation based on clinically meaningful intervals. These expert elicited intervals are as listed in Table 1.

Third, we developed a strategy to deal with missing data, which comprises 32% of LUCADA. Data incompleteness is a fact of life for clinical datasets [5,42] and depending on how the incompleteness of any particular variable is related to other variables, missing data is commonly modelled based on one of three different assumptions: 1) missing completely at random (MCAR); 2) missing at random (MAR); or 3) not missing at random (NMAR), where the latter comprises all those cases that do not fall under 1 or 2, and as such necessitates modelling missing data explicitly. 

The two common methods to deal with MAR data are Expectation Maximisation (EM) and Multiple Imputation (MI) [43]. However, it has to be borne in mind that both EM and MI are computationally complex algorithms that may not be feasible for large datasets with high rates of incompleteness. More importantly, their usage depends substantially on the validity of the MAR assumption, without which they result in biased estimates [44]. Graham advises that “the best way to think of all missing data is as a continuum between MAR and MNAR” and one has to decide whether the MAR violation in a given data set is big enough to render the estimates of MI and EM invalid [45].

Informed by our interactions with the NLCA staff, we concluded that NMAR missingness was prominent in LUCADA and the adoption of EM or MI could have negative effects. As a result we opted to model “missingness” explicitly given the context. In fact, missing data patterns in clinical datasets are often correlated with the clinical relevance of the missing values for a specific patient and may often embody information [42,46]. In order to evaluate whether or not the absence of data in the LUCADA data could provide useful information in building prediction models, we ran a set of experiments on our chosen 13-variable subset with 117,426 patient records. 

To this end, we chose 1-year survival as our binary outcome variable and separated the rest of the dataset as our prediction matrix. Following this, we prepared a binary ‘indicator matrix’ whose elements were zero or one depending on whether the corresponding elements of the prediction matrix were observed or were missing. We input the resulting indicator matrix into the Naïve Bayes [47] and Logistic Regression [47] algorithms and in each case predicted 1-year survival. The AUC values and predictive accuracy percentages achieved by the information on data incompleteness alone are given in Table 3. The values reported in the table are the averages and the standard deviations of 10-fold stratified cross-validation results. 

**Table 3:** Area under the curve (AUC) and predictive accuracy performance results for the missing data indicator matrix in predicting 1-year survival outcome.

	**Average AUC**	**Std. Dev. AUC**	**Average Accuracy**	**Std. Dev. Accuracy**
**Logistic Regression**	0.72	0.024	72	0.37
**Naive Bayes**	0.69	0.021	71	0.36

These results clearly show that the missing data pattern is actually highly informative in predicting 1-year survival in the LUCADA dataset. For this reason, we opted to model missing data explicitly in our analyses. In doing so, we used PostgreSQL[48] queries to replace the null observations in the database with an explicit “Unknown/Missing” state.

## Experimental Methods

The applicability of BNs to predicting 1-year survival in the LUCADA dataset was motivated above. Structure learning of the associated DAGs can be carried out manually or, in the presence of a comprehensive data set, via automatic causal discovery algorithms. In our experiments, we compared the plausibility of the DAG structures, which were 1) elicited from the clinicians’ perception of the domain; 2) learned strictly from data; and 3) learned via a hybrid approach that incorporates the expert knowledge into automated structure learning.

Expert elicited structures are very common in clinical applications, since the causal relationships between different variables are well understood by clinicians. Lucas et al. report that many of the BNs [28,49–55] developed for real life applications in biomedicine and healthcare have been constructed manually [4]. However, such BNs are prone to subjective biases and may not be able to fully capture statistical signatures (such as independencies) that are implicit in the data. These may result in suboptimal models, especially in cases where the end goal is posterior parameter estimation or classification, rather than making explicit the causal relationships to gain a better understanding of the problem domain.

On the other hand, the automatic learning of the causal structure of a BN from data is an active challenge pursued in ML, particularly because there is no unique BN that represents the joint probability distribution given by the data [9]. In general, automatic structure learning algorithms can be categorised into: 1) Constraint-based algorithms that use conditional independencies; and 2) Score-based search algorithms, which search for the DAG model that maximises a metric score in the causal model space [13]. The constraint based methods are focused on recovering a causal structure based on conditional independencies in the data. In our experiments we made use of an improved version of Inferred Causation (IC) algorithm as described in [56] and implemented by Bouckaert in WEKA 3 [57].

The score-based search algorithms make use of decomposable scores that allow the total score for a DAG to be calculated as the sum (or product) of the individual node scores in the network. In our experiments, we made use of the K2 score [58], which is a type of Bayesian score [58–60], in order to calculate the joint probability of a graph (G) and the dataset (D) [58]. The general equation for a Bayesian score is given in equation 1.


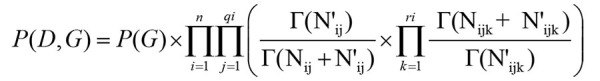
(1)

All automated learning algorithms presented in this paper were implemented either in the MatLab BNT toolbox [61] or the WEKA 3 [57] machine learning software. Specifically, in our experiments we used the following score-based search algorithms: 1) Tree Augmented Naïve Bayes (TAN), which was introduced by Friedman and Geiger as a relaxation of the strong independence assumption between the predictor variables in a Naïve Bayes classifier [62]. The version of TAN that we used was implemented in WEKA 3; 2) K2, which was proposed by [58] and implemented in the BNT toolbox; 3) Markov Chain Monte Carlo Model Decomposition MC^3^, first proposed by Madigan and York [63] and implemented in the BNT toolbox; and finally 4) Simulated Annealing for searching the space of all probability models, as implemented by Bouckaert in WEKA 3 [57].

In addition to these fully automated algorithms, we also explored the use of a hybrid structure learning algorithm, named Causal Minimum Message Length (CaMML) [64], which enables different types of expert knowledge, such as temporal tiers (A happens before B, denoted as A ≺ B), direct relations (A and B are related, denoted as A − B) and direct causal connections (A directly influences B, denoted as A → B), to be incorporated into the automated learning process. For structure learning, we used the Java implementation of CaMML, developed at Monash University. It has previously been used by Flores et al. [15] and Twardy et al. [65] to learn clinical causal structures in the domain of cardiovascular disease. Overall, a common attribute of all the structure learning algorithms used was that they assumed all variables to be discrete and the dataset to be fully observed. 

### Experimental Setup

In all of the BN experiments, we represented the joint probability distributions using conditional probability tables (CPTs), which were learned via maximum likelihood estimations by assuming uniform Dirichlet prior distributions over all discrete variables. This “levelled the playing field” in terms of parameterisation. We focused our efforts on comparing the variation of the structure learning algorithms.

We carried out all experiments by partitioning the selected 117,426-patient-strong subset of LUCADA into 10 equally-sized parts with approximately equal prior outcome probabilities, where probability of 1-year survival was 0.33. For each BN experiment, structure and parameter learning were performed on 9 partitions and tested on the remaining one. By iterating this process over all ten partitions, we ensured the inclusion of all patient records in the experiments. The performances of all causal BNs and other predictive models were evaluated based on the AUC values and predictive accuracy percentages of these stratified ten-fold cross-validations. 

**Figure 1:**
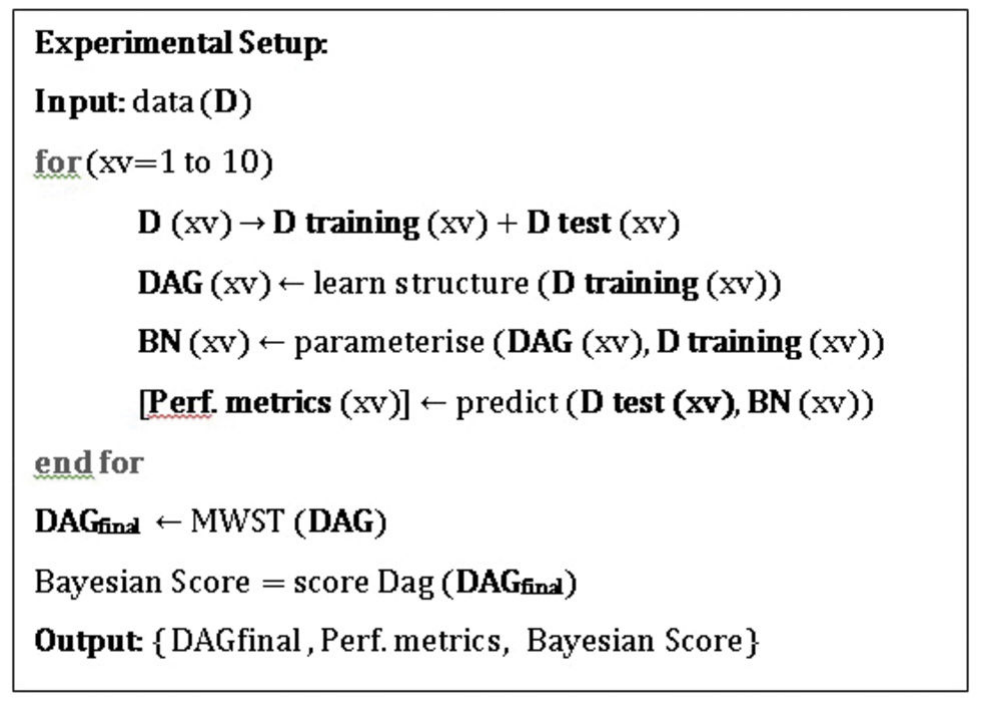
The experimental setup for structure learning. The pseudo-code of the experimental setup for learning and assessing DAGs via different algorithms.

The experimental set-up by which we learned the structure and parameters and report predictive performance metrics with each algorithm is summarised in Figure 1. For each fold of cross-validation, we separated the dataset D(xv) into training and test sets. We used the training set to learn the DAG and parameters of the BN, and then the test set to evaluate the predictive performance of the learned structure. According to this, we represented the **DAG** (xv) for each fold in the form of a logical adjacency matrix. At the end of the cross validation, we input the **DAG** array, which consisted of all structures learned during the 10-fold cross validation, into a directed maximum spanning tree (MWST) algorithm in order to acquire the resulting **DAG_final_**. We then made use of the Bayesian Score metric, given in Equation 1, in order to calculate P (**D**, **DAG_final_**).

Though our main focus is on BNs, in order to provide baseline reference benchmarks, we also report classification performances obtained by the widely used Naïve Bayes (NB), Logistic Regression, and the C4.5 decision tree algorithm. In our experiments, we made use of the NB algorithm in MatLab R2011a. For Logistic Regression and the C4.5 decision tree algorithms we used WEKA 3 [66]. NB has been adopted as the baseline performance metric in many ML studies. Despite its simplicity, it has been reported to yield comparable results to more sophisticated ML techniques, especially in the presence of large datasets [67,68]. Logistic regression is commonly used in clinical cohort studies and trials [69]. The specific implementation of Logistic Regression in WEKA 3 is based on using ‘ridge estimators’ for improving coefficient estimates [70]. C4.5 is a commonly used algorithm for building decision trees, which are deemed to be particularly suitable for domains with discrete variables like ours [71,72]. The specific implementation of the C4.5 algorithm that we used in WEKA 3 is named “J48”.

### Inference

As emphasised earlier, one of our reasons to represent our domain as a BN is the versatility of probabilistic inference provided by BNs, whereby entering evidence on any variable in the network results in updating the posterior distributions of the rest of the variables. These probability updates, i.e. belief updates, can be visualised on top of the graph structures, providing a degree of transparency during inference. This differentiates BN inference from “black-box” ML processes [9]. 

**Figure 2:**
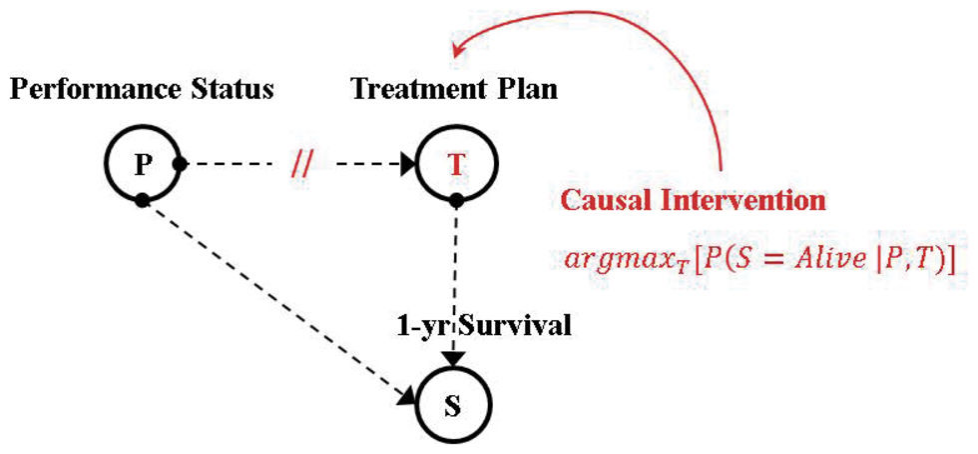
Schematic explanation of a causal intervention on a BN.

In all our experimental results, we made use of the Junction Tree algorithm [73] as separately implemented by Murphy [61] in the MatLab BNT toolbox and by Bouckaert [57] in WEKA 3. This algorithm consists of ‘moralising’ and ‘triangulating’ a DAG structure to create a junction tree structure over which a message passing algorithm is run for belief updating. The usage of such a message passing algorithm has certain implications in belief updating in causal interventions [9]. This can be explained with a context-specific example as given in Figure 2.

The message passing algorithm operates through forward and backward propagation of observed evidence in the graph. As a result, when we intervene manually on T, as shown in Figure 2, Pearl suggests that all edges from the parents of T to T need to be removed in order to eliminate the indirect path connecting T to S through P [8]. Put more simply, the direct intervention on T should render the effects of all parents of T on T ineffective; we illustrate this in Figure 2 with the edge from P to T “cut”.

## Results

### BN structures learned from LUCADA

**Figure 3:**
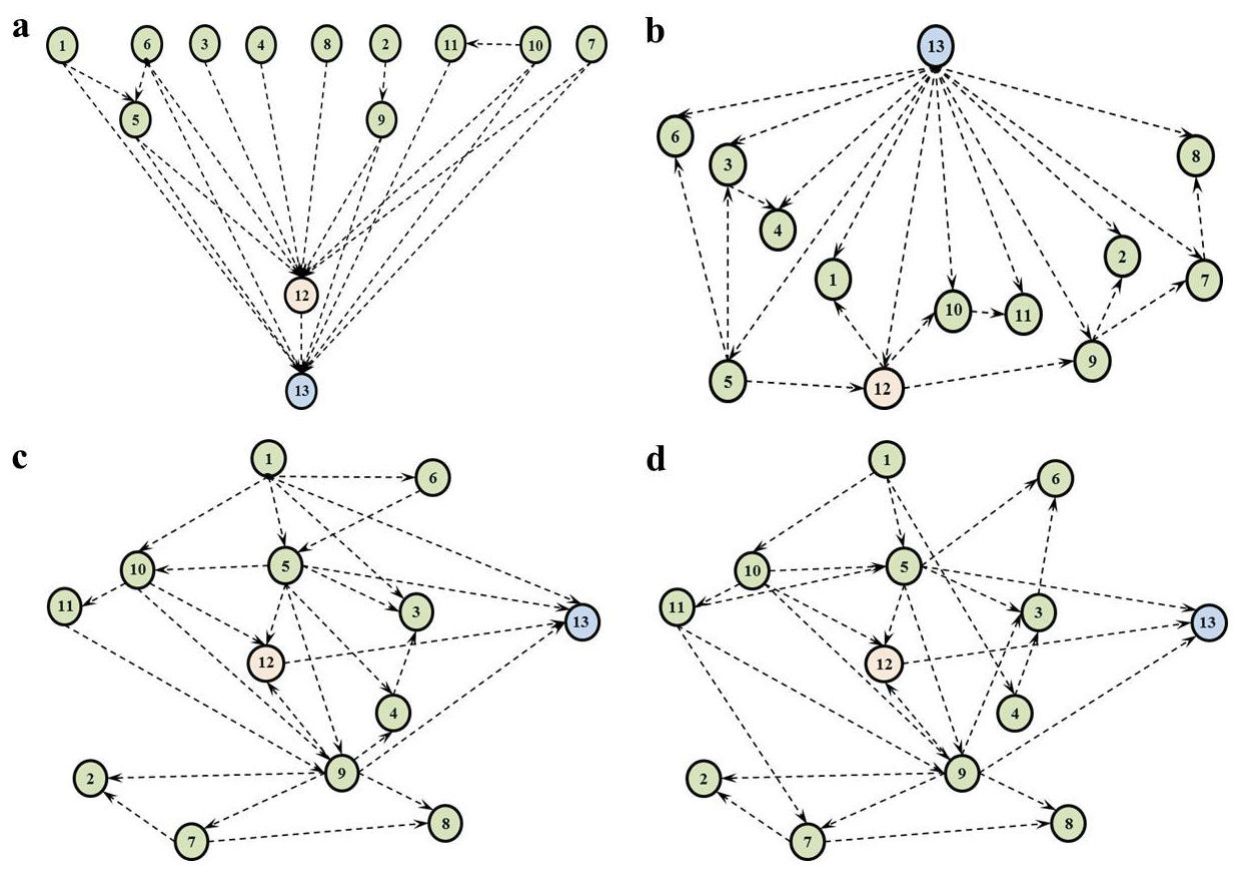
DAG structures learned by different methods. (a) manual construction, (b) Tree Augmented Naive Bayes (TAN) algorithm, (c) CaMML algorithm with structural pair-wise priors, (d) CaMML algorithm with temporal tiers.

In order to discover a structure that encapsulates the causal domain knowledge of the clinicians, while achieving a high Bayesian score and predictive performance, we tried various causal discovery approaches. First, without any algorithmic aid, we elicited the causal structure of the domain with the help of our clinical collaborators. This structure, given in Figure 3a, was built by asking the clinicians to connect the 13 domain variables based on a notion of causality; more specifically, asking them to point out the direct influences each variable has on others. As can be seen in Figure 3a, there is limited interaction between the pre-treatment variables (1-11) and the edges often point from the pre-treatment variables to the ‘Suggested Cancer Treatment Plan’ (12) and ‘1-year Survival’ (13) variables. 

**Figure 4:**
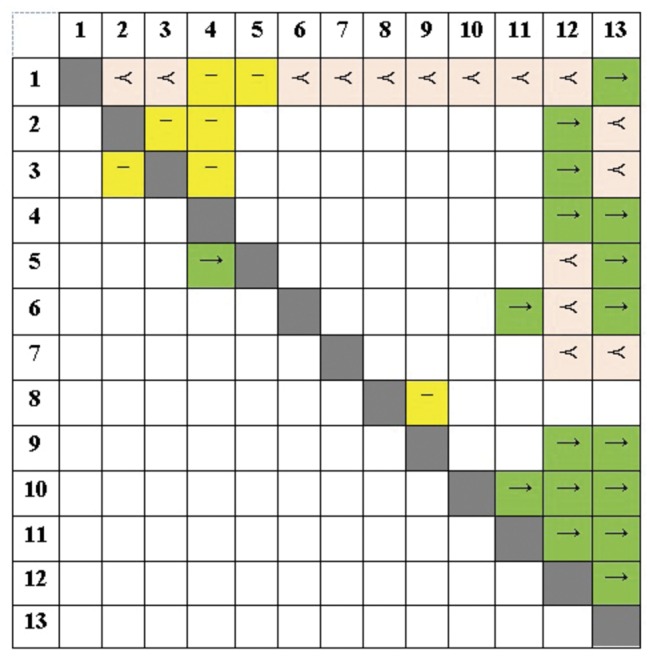
Expert elicited structural pairwise relations based on the selected 13 LUCADA variables. The variable codes are as given in Table 1. The notations can be read as: “A≺B”: A happens before B; “A−B”: A and B are related; and “A→B”: A influences B.

During knowledge elicitation, we also gathered different types of pairwise relational information from the clinicians in order to use for hybrid learning with CaMML. As can be seen in Figure 4, the direct causal influences (“A→B”) of the pre-treatment variables on the treatment selection and treatment outcome variables are also prevalent in this pairwise-relations matrix. However, the flexibility of defining additional relation types as undirected relations (“A−B”) and temporal orders (“A≺B”) yields a slightly different view of the domain. Overall, we ran three experiments with CaMML that used 1) no expert knowledge; 2) temporal tiers information as colour-coded in Table 1; and 3) structural pairwise relations given in Figure 4. We used confidence levels of 1.0 and 0.8 for the temporal tier and pair-wise relationship experiments, respectively.

Following these, we ran the automated learning algorithms listed in the previous section in order to learn the structures directly from the data. The results of our experimental runs, which reflect the Bayesian scores and predictive performances achieved by different learning approaches, are given in Table 4. To serve as a reference, in the same Table we also include the performances of the three baseline benchmark algorithms on predicting the “1-yr Survival” outcome. 

**Table 4:** Predictive performance metrics and Bayesian Scores for the 10-fold stratified cross validation experiments with the corresponding algorithms. The AUC and Accuracy % columns represent the means and standard deviations of the cross validated results.

	**AUC**	**Accuracy %**	**Log Bayesian Score DAG_final_**
**Logistic Regression**	0.812 (±0.04)	77.00 (±0.61)	-
**C4.5**	0.767 (±0.04)	75.64 (±0.59)	-
**Naïve Bayes**	0.793 (±0.04)	75.04 (±0.67)	**-**
**Tree augmented Naïve Bayes**	0.810 (±0.05)	76.93 (±0.63)	-1,558,894
**BN, IC**	0.793 (±0.04)	75.04 (±0.74)	-1,763,061
**BN, K2**	0.809 (±0.04)	76.49 (±0.69)	-1,590,874
**BN, Simulated Annealing**	0.807 (±0.04)	76.50 (±0.71)	-1,567,118
**BN, MCMC**	0.807 (±0.05)	74.24 (±0.54)	-1,600,891
**BN, CaMML - no priors**	0.806 (±0.03)	73.10 (±0.73)	-1,586,574
**BN, CaMML - temporal tiers**	0.806 (±0.03)	74.31 (±0.72)	-1,570,878
**BN, CaMML - structural priors**	0.805 (±0.03)	74.27 (±0.64)	-1,581,243
**BN, manually built structure**	0.749 (±0.03)	68.30 (±0.62)	-2,093,036

Focussing initially on the average predictive performances listed in Table 4, we see that Logistic Regression and the TAN algorithm achieve marginally higher AUC (81%) and predictive accuracy (0.77) results. However, the results achieved by these two algorithms are not statistically different from the rest. Overall, the performances of the reference benchmark algorithms and the BNs are quite similar. The two exceptions to this are 1) the decision tree learned by the C4.5 algorithm, which achieves a low AUC value relative to all other algorithms; and 2) the manually elicited structure given in Figure 3a, which obtains the worst classification performance among all. Although it may be intuitive to expect that the dependencies as perceived by the domain experts should be more robust compared to those that are learned from a dataset of limited size, this low predictive performance of the manual DAG structure may be explained by implicit dependencies in the data that the clinically elicited network is unable to capture.

Furthermore, from Table 4 it is also evident that the manually elicited structure attained the lowest Bayesian score among all others. On the other hand, the structure that obtained the highest Bayesian score is the one learned via the TAN algorithm in Figure 3b. It should be noted that despite our inclusion of TAN among our structure learning algorithms, as a slight relaxation of NB, it is not intended for causal discovery. 

Despite operating to maximise a different metric score, namely Maximum Message Length (MML), the structures learned by CaMML achieve comparable Bayesian scores to the other score-based search algorithms. The structures learned by CaMML by incorporating the pair-wise relationships and the temporal tiers are as given in Figures 3c and 3d, respectively. Compared to the manually built structure (Figure 3a), these are less connected and have lower number of parents directly pointing to the treatment selection (12) and survival (13) variables. Examining these structures and their corresponding rows in Table 4, we can see that while the incorporation of expert knowledge into the learning process has little effect on the Bayesian score or the predictive performances attained, it helps yield structures that look more similar to the expert elicited structure given in Figure 3a. 

This is an important feature that meets our criteria of uncovering the most feasible causal structure that has a high probability given the data, while being in line with -or at least not openly violating- the causal understanding of the domain as perceived by the clinicians. For this reason, we chose the causal structure learned based on the CaMML temporal tiers (Figure 3d), since it achieves the highest Bayesian Score among other structures that take into account expert knowledge. We used this structure in our causal intervention experiments, the results of which are presented in the next section. 

### Effects of Treatment Selection on Survival

**Figure 5:**
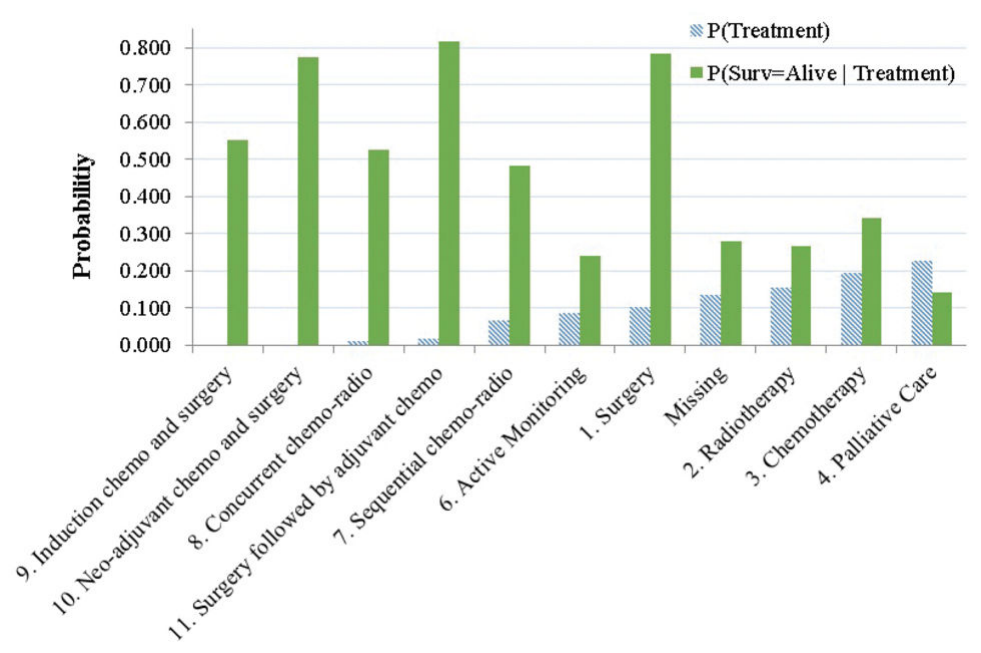
The treatment plan probabilities as calculated from LUCADA. P(Treatment) is represented in striped blue columns, and the conditional 1-year survival probabilities given specific treatment plans, P(Surv = Alive│Treatment), is represented in green columns The horizontal axis, which contains the treatment plan options, is ordered in descending order of P(Treatment) from left to right.

After selecting the BN structure, we set out to investigate whether it could be used for making plausible treatment recommendations based on the interventional query of “P(Survival=Alive | Evidence, T)= ?”. As a prelude, we investigated: 1) the probabilities of treatment plans: P(Treatment); and 2) the conditional probabilities of 1-year survivals when a specific treatment plan was given: P(Survival=Alive |Treatment), as observed in LUCADA. Figure 5 shows these treatment probabilities and the 1-year-survival probabilities conditional on treatment plans as (striped) blue and green columns respectively for each treatment plan. The lack of correlation between the treatment frequencies and conditional survival probabilities in Figure 5 may reflect the fact that survival maximisation is not the only parameter affecting the eligibility of the patients for a particular treatment plan. We elaborate further on this while discussing our results. 

The discrepancy between P(Treatment) and P(Survival =Alive |Treatment) is more pronounced in treatment plans that involve surgery (1, 9, 10, 11 in Figure 5). For instance, focusing on ‘11.Surgery followed by adjuvant chemotherapy’, we observe that P(Survival=Alive│Treatment=11)=0.81, while P(Treatment=11)=0.02. This means that despite the high chances of survival if given the treatment, the joint probability, P(Survival=Alive, Treatment=11), of observing a patient, who has been given ‘Surgery followed by adjuvant chemotherapy’ and survived at least one year, is relatively low at a meagre (0.81×0.02)=0.016 in the database. 

Our primary motivation was to compare the concordances of the recorded treatment plans with the BN treatment recommendations based on survival maximisation. We evaluated concordance with respect both to exact and partial matches between the top system recommendations and the recorded treatments for a carefully selected subset of LUCADA. This subset only contained patients who: 1) were diagnosed with SCLC or NSCLC; 2) were given a curative treatment plan; and 3) had no missing data. This resulted in a fully observed patient subset of 4020 patients. In addition, we excluded from our causal interventions the non-curative treatment plans, namely ‘Active Monitoring’ and ‘Palliative Care’. 

**Figure 6:**
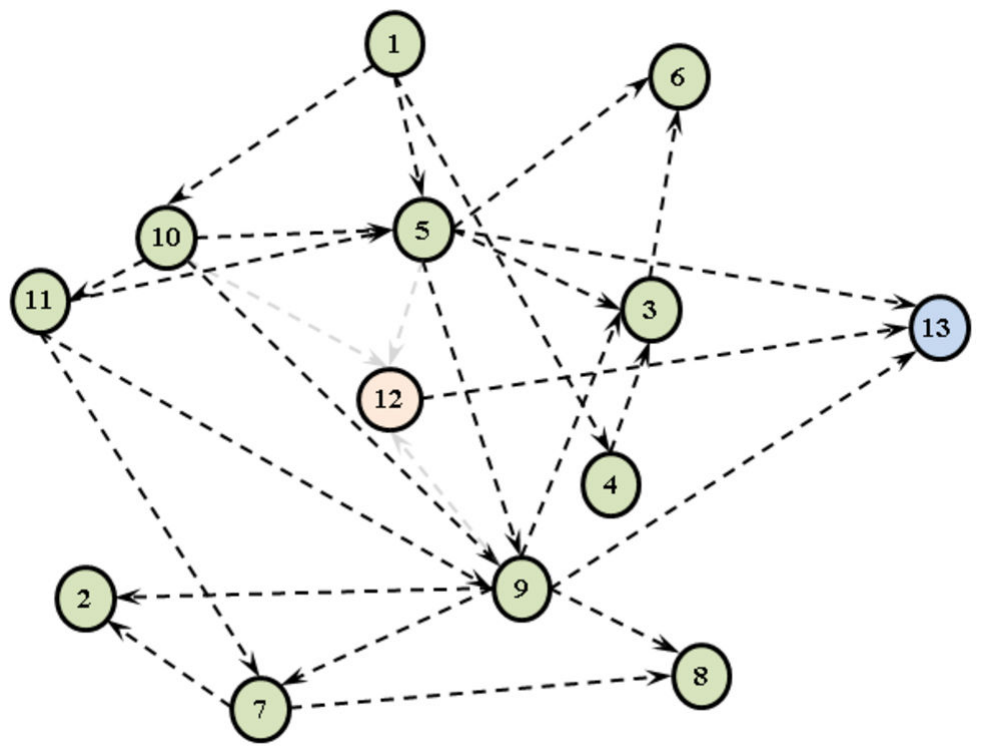
The modified BN Structure for causal interventions. All edges towards the intervened “Suggested Cancer Treatment Plan” are removed.

Furthermore, before running the causal interventions, we modified our chosen DAG structure (Figure 3d) by removing the edges directed at the intervened “Suggested Cancer Treatment Plan” (12) variable, as recommended by Pearl [8]. The resulting DAG is shown in Figure 6. We re-parameterised this modified BN, excluding the 4020-patient strong subset that we set aside to assess the plausibility of survival-based causal intervention queries.

Overall, the percentage of patients for whom there was an exact concordance between the top BN recommendation, argmax(T)[P(Survival=Alive |Evidence, T)], and the recorded treatment was very low at 29%. However, and crucially, this percentage rose to 76% when we included partial matches between the two. An example of a partial match is where the recorded treatment plan is ‘Surgery’, while the top BN recommendation is ‘Surgery followed by adjuvant chemotherapy’.

## Concordances with Respect to Recorded Cancer Treatment Plans

**Figure 7:**
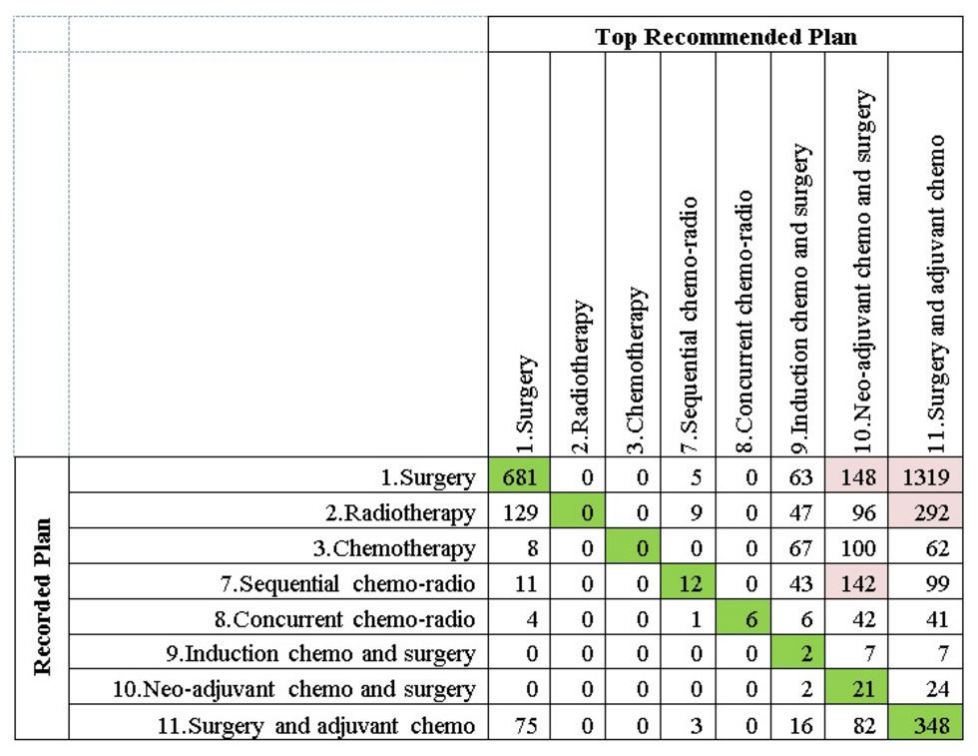
The confusion matrix that displays the recorded versus the recommended treatment plans. The recommended treatment plans are the ones that maximise 1-year survival for a patient, acquired via causal intervention on the BN.

We analysed the level of exact and partial concordances with respect to the recorded treatment plan types in the dataset. Figure 7 is a confusion matrix summarising the aggregated discrepancies between the recorded treatment plans in the dataset and the top recommendations provided by the BN. The highlighted non-diagonal cells represent the most prevalent sources of discordance between the recorded treatments and the top BN recommendations.

A pattern that is clearly visible in Figure 7 is that the top treatment recommendations by the BN almost exclusively comprise surgery (labelled as 1, 9, 10, and 11). If we focus on the non-surgical treatment plan columns (labelled as 2, 3, 7, and 8) we see that the single modality plans: radiotherapy and chemotherapy are never recommended by the system, and the multimodal chemo-radiotherapy plans are recommended very rarely. 

Focusing on the ‘Surgery’ row in Figure 7, we see that for the majority of the cases, the BN favours multimodal surgical treatment plans: 9, 10, and 11 over surgery alone. Analysing the characteristics of the 681 concordant cases, we found that these were all early stage patients for whom surgery alone yielded marginally better survival likelihoods compared to the multimodal surgical plans. Another interesting observation is that the treatment plans 9 and 10 are on-going clinical trials and are currently only given to a limited number of patients. As can be seen in the confusion matrix, based on maximising the probability of 1-year survival, the BN recommends these plans for a significant number of patients. 

**Figure 8:**
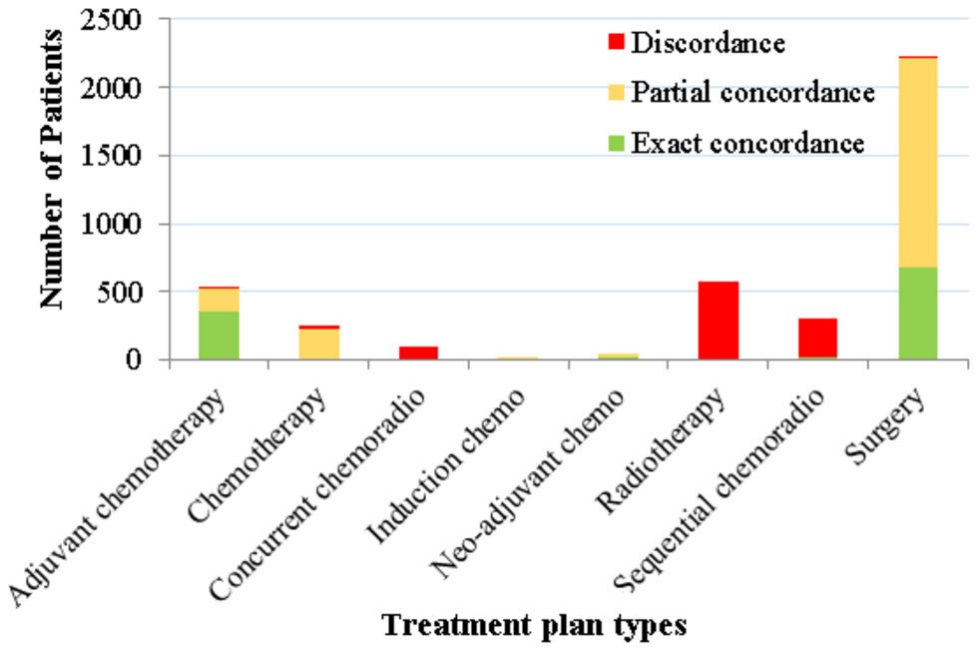
The exact and partial concordances between the recommended and the recorded treatment plans. The concordances are stratified with respect to treatment plan types. The recommended treatment plans are the ones that maximise 1-year survival for a patient, acquired via causal intervention on the BN.

In addition to the confusion matrix in Figure 7, Figure 8 is a stacked column graph that summarises the exact and partial concordances with respect to different treatment plan types. Concentrating on the non-surgical treatment columns, we observe that they mostly contain discordant cases. 

**Figure 9:**
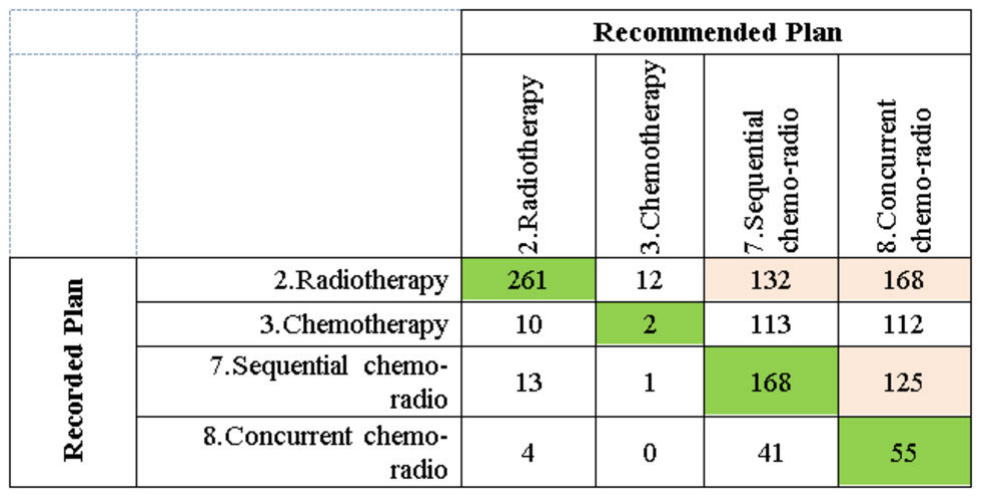
The non-surgical confusion matrix for patients who have been treated with non-surgical treatment plans. The recommended treatment plans are the ones that maximise 1-year survival for a patient, acquired via causal intervention on the BN.

It is clear both from Figures 7 and 8 that the maximum a posteriori (MAP) estimations of argmax(T)[P(Survival=Alive |Evidence, T)] produce recommendations that are heavily biased towards surgical treatment plans. For this reason, we carried out a second set of experiments in which we only included those patients from the selected subset for whom the recorded treatment plan was non-surgical. Furthermore, in order to assess whether the concordance levels improved when we manually eliminated surgical treatment plans as viable options, we excluded surgical treatment plan types 1, 9, 10 and 11 from our interventions. Figure 9 shows that when surgical treatment plans are discarded, the exact concordance levels between the system recommendations and recorded treatments increase substantially.

When we investigated the characteristics of the concordant and discordant cases on the ‘Radiotherapy’ row, we found that the 161 concordant cases were all early-stage (IA to IIB) cancer patients, for whom the 1-year survival probabilities achieved by ‘Radiotherapy’ alone are marginally higher than the multi-modal treatment plans 7 and 8. However, from stage IIB and upwards, the BN recommendations heavily favour multimodal chemo-radiotherapy treatment plans 7 and 8 over radiotherapy alone.

Further analysis of the posterior survival distributions for the patients, who were recommended either sequential or concurrent chemo-radiotherapy plans (7 and 8) according to survival-maximisation, showed that in most cases the 1-year survival probabilities with either treatment plan were very similar, slightly varying in favour of 7 or 8 depending on patient characteristics. The system’s inability to distinguish between these two plans may be indicative of additional criteria, other than maximising survival, that affect this decision in real life. Finally, a striking observation in Figure 9 is that, apart from 2 patients (who were both stage IIA), the causal non-surgical treatment plan interventions on the BN never resulted in ‘Chemotherapy’ being the treatment that maximises survival. As is evident, in the majority of such cases, the system favoured the multi-modal chemo-radiotherapy plans over chemotherapy alone. 

## Concordances with Respect to TNM Stages

**Figure 10:**
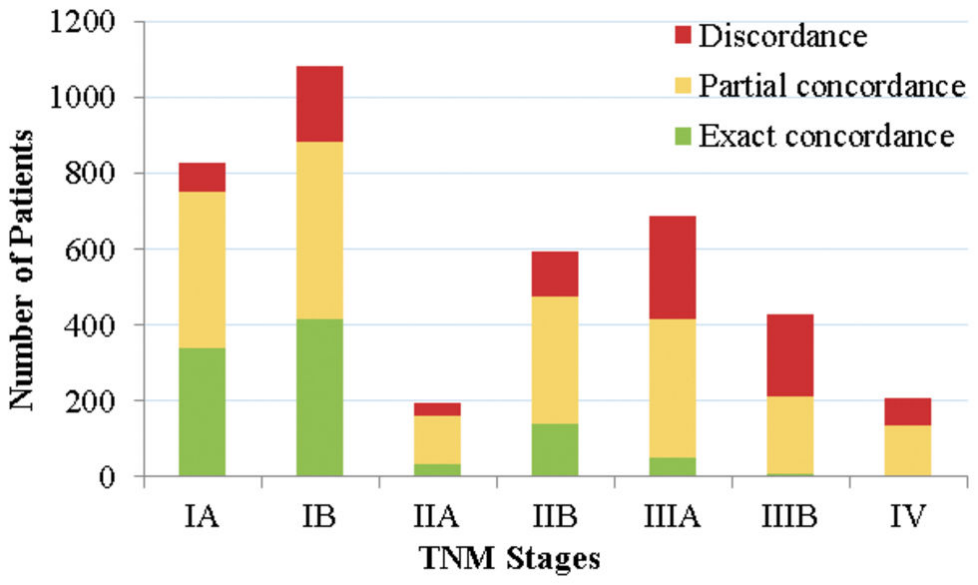
The exact and partial concordances between the recommended and the recorded treatment plans. The concordances are stratified with respect to the TNM stages.

In addition to our analyses of concordance based on treatment plan types, we also investigated the levels of exact and partial concordances with respect to the TNM stages of the test patients, as plotted in Figure 10.

As can be seen, the exact concordance levels plummet for locally advanced (IIIA and IIB) and advanced (IV) stage patients. This may be explained by the fact that in clinical practice, the proportion of patients who are suitable for surgery decreases as the severity of the disease (judged by the TNM stage) increases. However, in contrast, the BN favours surgical treatment plans regardless of the stage of the disease. These results indicate that the BN treatment recommendations, which are solely based on maximising survival, do not agree with the recorded treatment plans in general. 

## Discussion

This study presents an evaluation of the feasibility of BNs in providing accurate personalised survival estimates and treatment selection recommendations based on the effects of different treatment plan options on this estimated survival. The empirical results are based on various patient and disease-specific variables selected from a large national lung cancer patient dataset, namely LUCADA. In order to obtain a plausible causal structure that also achieves a high Bayesian score, we have compared the performances of manual structure elicitation to various automated and hybrid causal discovery algorithms.

Consistent with the findings of Forsberg et al. [7] and Stojadinovic et al. [16], our survival prediction results indicate that BNs are both viable and robust inference tools for predicting patient survival outcomes with high predictive accuracy and AUC rates. However, we also found that the predictive performances achieved by the BNs do not offer a significant improvement over, but are comparable to, those resulting from less complex classifier algorithms as Naïve Bayes, Logistic Regression or C4.5 decision trees. 

Despite not providing a significant improvement in prediction performance, we contend that BNs still provide unique benefits pertaining to encoding of and reasoning with large patient cohort data. In particular, their suitability for carrying out causal interventions allows them to be utilised for answering complex clinical questions that are based on unobserved evidence. Another advantage of the probabilistic inference provided by the BNs is that the probability distributions underlying the network can be automatically updated to incorporate newly added patient information. This adaptive nature of the BNs allows building autonomous systems that can evolve as more data is added [74] . 

To date, we have experimented with the most prominent structure learning algorithms. The results presented herein are not sufficient to make a definitive statement about which structure learning methodology is the best, since different methodologies prioritise different factors -e.g. various score metrics, statistical dependencies, and expert opinion- for causal discovery. However, we found that different automated learning algorithms yield substantially different final structures. As a stark example from our results, despite their relatively similar Bayesian scores, there is actually a substantial difference between the DAGs learned by the CaMML and TAN algorithms, whereby the arc directions in the highest scoring TAN algorithm actually defy any temporal or causal pattern within the domain. This inability of the automated structure learning algorithms to yield a single DAG that faithfully represents the causal structure of a given domain is a well-known issue in causal discovery. Many causal discovery algorithms avoid dwelling on this issue, or presuppose some solution to the problem of identifying a correct variable order [9].

As an alternative to automated structure learning, we also elicited a manual structure from our clinical collaborators. As mentioned earlier, this approach is commonly used for BN applications in clinical domains. Our experimental results indicate that the manually constructed BN does not represent the best model in terms of either predictive performance or fit to data in our case. In practice, both automated and manual constructions of DAGs have limitations. As an alternative to these, hybrid causal learning is an emerging field and -as our results indicate- shows promise in making the most of the two separate approaches by obtaining causal structures that yield high performance metrics while retaining the causal patterns set out by domain experts. This allows a facilitated combination of domain knowledge and data, a key function that BNs are inherently suitable for. Our empirical results with the hybrid learning algorithm CaMML are a contribution to this developing field. 

Meanwhile, the causal intervention results, which compare the concordances between the recorded treatments and the recommended treatments by the BN, reveal that a posteriori estimations based on maximising 1-year survival are highly biased and therefore not reliable in predicting recorded lung cancer treatment plans on their own. The main source of disagreement between the top system recommendations and recorded treatment plans stems from the discrepancies between the conditional and joint probabilities of 1-year survival as reflected in Figure 5. Due to the nature of our causal interventions, the system recommendations are based on the conditional survival probabilities, while the frequencies in the database reflect the joint probabilities P(Survival=Alive, Treatment). The lack of correlation between the two indicates that survival maximisation is clearly not the only parameter affecting a patient’s eligibility for a treatment plan. 

In fact, there are various other factors that govern treatment selection decisions [6,29], as: 1) the suitability of the patient for surgery or other treatment modalities; 2) the quality of life evaluation during and after treatment; and 3) an economic analysis on the cost efficiency of the treatment plans. Despite their importance, the causal intervention queries in our experiments do not take these additional factors into account. This is primarily due to lack of available data on these factors in LUCADA, which prevented us from causally intervening on additional variables such as ‘suitability for resection (R)’ and ‘cost effectiveness (C)’, alongside 1-year survival (S). In the presence of relevant data, such interventions can be carried out more efficiently via Bayesian decision networks [75,76], which are generalisations of BNs with added functionality that allow multi-criteria decision and utility analyses. 

Although the maximum a posteriori estimations achieved by the causal interventions on the BN were not accurate in predicting the recorded treatments, embedding the posterior distributions returned by the BN within a decision support system and making them available to the clinicians may enable them to not only see what the treatment plan that would maximise survival is; but also to what extent it would improve survival expectancies relative to the alternative options. We are currently working on building such a system to be used in lung cancer MDT meetings. 

Overall, the results presented in this paper give sufficient encouragement to conduct more extensive experiments. Nevertheless, we recognise that the experiments reported here have a number of limitations. First, due to the lack of information (to this point) on 5-year survival rates, we have adopted a surrogate outcome measure, namely 1-year survival. Though this is justifiable [36,37], it is possible that the probabilistic treatment recommendations may eventually change when 5-year survival rates are used. 

Second, LUCADA, upon which we trained our BN and ran retrospective experiments on, contains treatment decisions which were supposedly arrived at by following clinical guideline rules. As such, it may reflect biased treatment patterns and survival rates. Unfortunately, the only systematic way of circumventing this inherent limitation would be by using data collected during prospective pilot studies, which span a minimum of 5-years and ideally involve randomised control groups.

Third, as the causal intervention results reveal, the system is not able to distinguish those patients eligible for surgery from those who are not. According to the British Thoracic Society and National Institute for Clinical Excellence guidelines, suitability for surgery should be determined by factors such as: risk of peri/post-operative mortality, cardiac functional capacity, lung function, and post-operative quality of life [6,29]. Unfortunately, we cannot incorporate these into our probabilistic queries since the relevant information is not available in LUCADA. Once the database is extended to store this information, the BN can be augmented with the addition of such variables and a “Suitability for Surgery” variable that would be a parent of “X-year Survival”, which can in turn be utilised to distinguish patients who are not suitable for surgery.
